# Development and Characterization of Novel Biopolymer Derived from *Abelmoschus esculentus* L. Extract and Its Antidiabetic Potential

**DOI:** 10.3390/molecules26123609

**Published:** 2021-06-12

**Authors:** Abd Elmoneim O. Elkhalifa, Eyad Al-Shammari, Mohd Adnan, Jerold C. Alcantara, Khalid Mehmood, Nagat Elzein Eltoum, Amir Mahgoub Awadelkareem, Mushtaq Ahmad Khan, Syed Amir Ashraf

**Affiliations:** 1 Department of Clinical Nutrition, College of Applied Medical Sciences, University of Hail, Hail P.O. Box 2440, Saudi Arabia; ao.abdalla@uoh.edu.sa (A.E.O.E.); eyadhealth@hotmail.com (E.A.-S.); nagacademic0509@gmail.com (N.E.E.); mahgoubamir22@gmail.com (A.M.A.); 2 Department of Biology, College of Science, University of Hail, Hail P.O. Box 2440, Saudi Arabia; drmohdadnan@gmail.com; 3 Department of Clinical Laboratory Sciences, College of Applied Medical Sciences, University of Hail, Hail P.O. Box 2240, Saudi Arabia; jerold.alcantara@yahoo.com; 4 Department of Pharmaceutics, College of Pharmacy, University of Hail, Hail P.O. Box 81481, Saudi Arabia; adckhalid@gmail.com; 5 Department of Microbiology and Immunology, College of Medicine and Health Sciences, UAE University, Al Ain 15551, United Arab Emirates; mushtaq.khan@uaeu.ac.ae

**Keywords:** okra mucilage, okra polysaccharides, biopolymer, α-amylase activity, α-glucosidase activity, nutraceuticals, antidiabetic activity

## Abstract

*Abelmoschus esculentus* (Okra) is an important vegetable crop, widely cultivated around the world due to its high nutritional significance along with several health benefits. Different parts of okra including its mucilage have been currently studied for its role in various therapeutic applications. Therefore, we aimed to develop and characterize the okra mucilage biopolymer (OMB) for its physicochemical properties as well as to evaluate its in vitro antidiabetic activity. The characterization of OMB using Fourier-transform infrared spectroscopy (FT-IR) revealed that okra mucilage containing polysaccharides lies in the bandwidth of 3279 and 1030 cm^−1^, which constitutes the fingerprint region of the spectrum. In addition, physicochemical parameters such as percentage yield, percentage solubility, and swelling index were found to be 2.66%, 96.9%, and 5, respectively. A mineral analysis of newly developed biopolymers showed a substantial amount of calcium (412 mg/100 g), potassium (418 mg/100 g), phosphorus (60 mg/100 g), iron (47 mg/100 g), zinc (16 mg/100 g), and sodium (9 mg/100 g). The significant antidiabetic potential of OMB was demonstrated using α-amylase and α-glucosidase enzyme inhibitory assay. Further investigations are required to explore the newly developed biopolymer for its toxicity, efficacy, and its possible utilization in food, nutraceutical, as well as pharmaceutical industries.

## 1. Introduction

*Abelmoschus esculentus* (L.) Moench is a popular vegetable crop cultivated throughout the world mostly in tropical and subtropical regions. The cultivation of okra vegetable is globally known for its palatability [1]. Okra plant and its derived products have been studied for various therapeutic purposes, such as antidiabetic, antioxidant, anticancer, immunomodulatory potentials, as well as its ability to ease constipation [1]. Currently, the mucilage or latex present in okra has drawn attention of the scientific community for its application as an intervention for new therapeutic purposes, as the infusion of okra mucilage has been earlier used in traditional Indian ethnomedicine for treating dysentery, diarrhea, and many more [2]. Previous studies indicated that okra mucilage could have a potential role in the management of diabetes [3]. Mucilaginous substances present in the pod walls of okra containing significant amount of protein, carbohydrate, neutral sugars, minerals, and other complex polysaccharides [4,5]. Polysaccharides are a very important class of biopolymers and represent a structurally diverse class of macromolecules. Furthermore, natural polymers derived from plant or animal sources have high molecular weight along with increased polarity, as these polymers are made up of monosaccharide units and joined by glycosides linkage [6]. Moreover, polysaccharides are highly diverse in structure and biological functions including serving as structural components of cell walls, cell recognition, cell proliferation, energy storage, cell differentiation, regulation of signaling, and immune responses [7]. This enormous potential variability in polysaccharide structure gives the necessary flexibility for the precise regulatory mechanisms of various cell–cell interactions in higher organisms. Recent research is focused on polysaccharides isolated from natural sources, because of its low side effect or with minimal toxicity. Meanwhile, several naturally occurring polysaccharides such as cellulose, starch, pectin, acacia gum, gum arabic, arabinogalactan, xylan, beta-glucan, and karaya gum has been reported [8,9]. Furthermore, the main bioactive component of okra mucilage is okra polysaccharide, which is reported to be comprising of pectic polysaccharides [10]. Meanwhile, the compositions of water-extractable polysaccharides were reported to be galacturonic acid, rhamnose, arabinose, xylose, mannose, galactose, glucose, xylan, starch, and uronic acid [11,12]. In addition, okra polysaccharides have been reported for their antioxidant activity, immunomodulatory activity, ability to improve metabolic disorders and intestinal function, hypoglycemic activity, and antifatigue activities. Rhamnogalacturonan, a polysaccharide extracted from okra, has been reported to have an antidiabetic effect [11]. 

Okra polysaccharides have been seen as a promising bioactive component considering its future prospective in food and pharmaceutical purposes for the development of novel polymer [10,11,13,14]. Additionally, okra polysaccharides could also become a source for the development of antidiabetic biopolymer, since it is considered to be very economical, non-toxic, and biodegradable [6]. Diabetes is currently one of the most prevalent epidemics worldwide; it represents an increase in socioeconomic burden, affecting about 382 million people globally, and each year, around 1.3 million people die from diabetes. By 2045, an estimate of 629 million people will be diabetic worldwide, as reported by the International Diabetes Federation in 2017 [15]. Moreover, the etiology of different types of diabetes varies, but complications related to high blood glucose are common in both types of diabetes. Meanwhile, drug or diet ability to delay the production or absorption of glucose by inhibiting carbohydrate-hydrolyzing enzymes such as α-amylase and α-glucosidase is one of the most common therapeutic approaches used for the treatment of hyperglycemia [16]. Additionally, α-glucosidase inhibitors are considered to be more effective categories of antidiabetic agents used in hyperglycemia, especially in case of postprandial hyperglycemia over α-amylase inhibitors. The membrane bound α-glucosidase enzymes speed up the digestion of oligosaccharides and disaccharides into simple monosaccharides, after which they get absorbed and enter into the bloodstream. The inhibition of α-glucosidase as well as α-amylases enzyme can help in delaying the digestion of carbohydrates, thereby reducing the levels of glucose in blood [17]. At present, the use of carbohydrate digesting enzyme inhibitors plays a vital role in controlling hyperglycemia by reducing the intestinal absorption of glucose [16]. 

However, several reports suggest that pharmacological agents are usually associated with some side effects, adverse effects, and even sometimes, their efficacies are controversial. Hence, attention has been shifted toward traditional and alternative medicines or food-derived products rich in antidiabetic phytoconstituents. The bioactive components present in plants and plants derived products such as alkaloids, flavonoids, glycosides, gum, carbohydrates, triterpenes, and different types of peptides are usually responsible for their therapeutic importance [18,19,20,21]. Recently, okra has been recognized for its potential therapeutic purposes due to the presence of various important phytochemicals including polysaccharides [1,22]. Therefore, despite having various therapeutic applications of okra fruits, seeds, pods, its mucilage has not been much explored toward its promising potential. Hence, a novel biopolymer derived from okra mucilage has been developed along with its physicochemical characterization, and its antidiabetic properties are studied.

## 2. Results and Discussion

### 2.1. Characterization of Okra Mucilage Biopolymer by FT-IR

**Figure 1:**
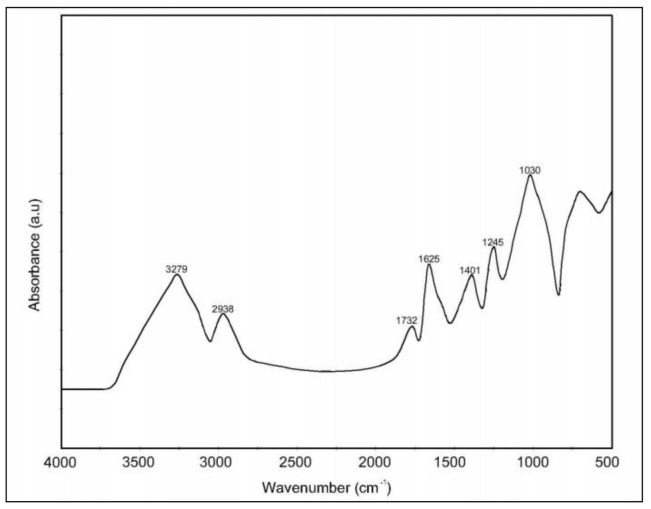
FT-IR spectrum of okra mucilage.

Okra mucilage biopolymer (OMB) characterization using FT-IR analysis identifies several functional groups representing characteristic bands of polysaccharides lying between 3279 and 1030 cm^−1^, which constitute the fingerprint region of the spectrum, as presented in Figure 1. The broad band at 3279 cm^−1^ is mainly due to the presence of hydrogen-bonded hydroxyl groups, which give rise to the complex vibrational bands associated with free intermolecular and intramolecular bound hydroxyl groups, which leads to the gross structure of carbohydrates [23,24]. Our FT-IR data also revealed the characteristic of polysaccharide consisting of galactose, rhamnose, and galacturonic acid represented by the broad-spectrum peak at 3279 cm^−1^, suggesting the presence of aromatic sugar with O-H as the principle functional groups. Meanwhile, the presence of an O-H functional group in the broad peak characterizes hydrophilic nature of the polysaccharides. The hydroxyl groups in carbohydrate have intermolecular and intramolecular hydrogen bonding that give broad band at 3279 cm^−1^ [24]. In addition, the band present at 2938 cm^−1^ is also a characteristic of methyl C-H bonding associated with benzene rings. In cellulose and hemicellulose components, the characteristic C–H stretching corresponds to the band at 2942 cm^−1^ [25]. In complex polysaccharides spectra, 1245 cm^−1^ was assigned for the C–O stretching band, whereas 1030 cm^−1^ was assigned for the C–O–C group, indicating the presence of aromatic bonds present in galactose, galacturonic acid, as well as in rhamnose. 

The amide I band corresponds to the band at 1625 cm^−1^, which comes in the most sensitive spectral region, depicting the secondary organizational units of proteins [26]. This band is purely due to peptide linkages and CO stretch vibrations, indicating the presence of protein. As the biopolymer was not subject to deproteinization, the protein bands were observed [27]. The absorption at 1732 cm^−1^ was observed because of ester carbonyl, which has also been reported in a previous study on okra mucilage [28]. Moreover, the presence of carbonyl, methyl, as well as hydroxyl functional groups in okra are representative of polysaccharides molecules, which is considered to be the backbone of the developed polymer. Our results confirmed that OMB is composed of polysaccharides. However, the polysaccharides were not in the form of cellulose or starch, but few functional bands indicated the presence of peptide cross-link along with some amino sugars, and our results are in line with earlier studies [24,29].

### 2.2. Percentage Yield, Solubility, and Swelling Index of Okra Mucilage Biopolymer 

Okra mucilage extraction and its percentage yield were found to be 2.66% *w/w* using water as the extraction liquid, and according to previous studies, the percentage yield of okra mucilage was reported to range from 0.5% to 11% [28,30,31]. These variations in the percentage yield of okra mucilage could be due to several factors, such as the physical state of pods (dried or hydrated), cultivation region of pods, breed of okra, parts of okra (crown or pulp), and maturation state of okra pods. In addition, the percentage yield could also be affected by the extraction method used [25]. It has been reported that acetone is more effective when compared to methanol as an extraction solvent; the yield rises by 31 times [28]. Percentage yield was recorded on a dry weight basis, and the moisture content of extracted mucilage was found to be 9.6%. Currently, scientific communities are working more on these factors to determine the optimal conditions of mucilage extraction, such as extraction time, temperature, extraction cycle number, and raw material-to-solvent ratio. Okra mucilage extraction by the water extraction method gave the yield of 2.66%, and the conditions were temperature of 70 °C for 2 h and agitating at 200 rpm [32]. The solution obtained was caramel-colored, viscous, and slippery. We found that the solubility of extracted mucilage polymer at neutral pH was 96.9%. Earlier studies have also indicated that okra mucilage is partially soluble in cold water and soluble in warm water. Our results are also consistent with the previous reported studies. All the characteristics obtained were similar to those reported previously [31,33]. 

**Figure 2:**
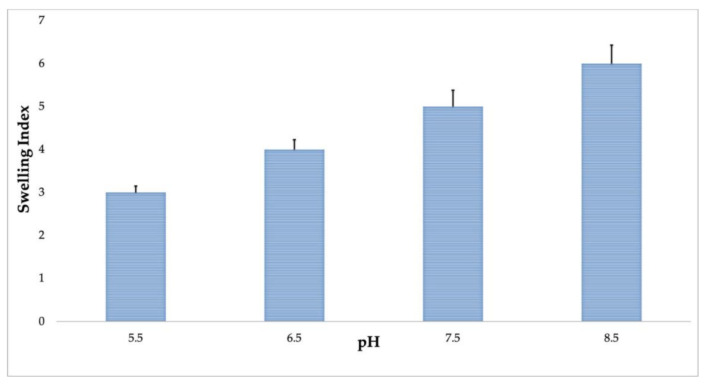
Swelling index of okra mucilage biopolymer at different pH. All experiments were performed in triplicate, and data represent mean ±standard deviation.

Additionally, we found that the swelling index of OMB at pH 7.5 in deionized water was 5.0. Moreover, the swelling index has been found to be increasing with a gradual increase of pH, as presented in Figure 2. This increase in the swelling index of okra mucilage could be due to its increase in intra-ionic repulson when the ionization of carboxyl groups (-COOH) is high. In addition, we also found that at pH 5.5, the swelling index was the lowest; this could be due to the low ionization of the major functional groups present in mucilage biopolymer. The pH-responsive behavior shown by OMB could present a favorable condition for the controlled release of a bioactive active component in pharmaceutical as well as nutraceutical industries [24]. In addition, the controlled release of active agents at different pH indicates a unique polymer behavior, which is indispensable in the packaging of food. Earlier studies have indicated that the polymer prepared for drug development shows reduced swelling onwards of pH > 7.4, which could be due to carboxyl groups ionization, and polymer dissolution occurs [34]. However, further studies are required for a better analysis of this reported behavior.

### 2.3. Mineral Composition

**Figure 3:**
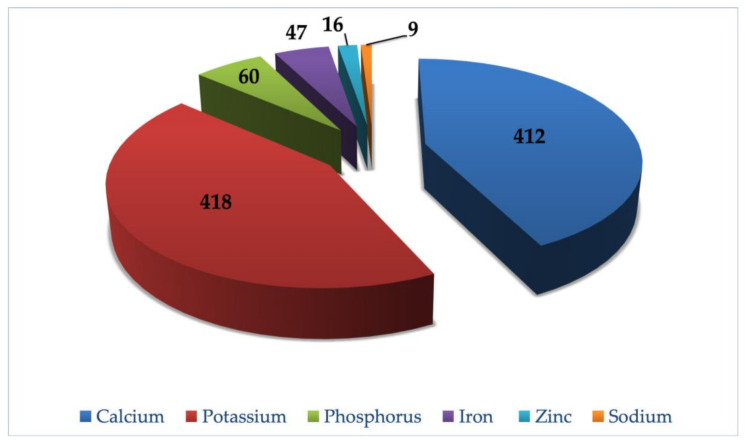
Mineral contents (mg/100 g) of okra mucilage biopolymer.

Minerals are considered as one of the important parts of human nutrition, which help to promote a healthy physical and mental state. The minerals are the principal elements of the bones, teeth, tissues, muscles, blood, and nerve cells [35]. They play an important role in acid–base balance maintenance, nerve response to physiological stimulation, and in the clotting of blood [36]. The major component of bone is calcium, which is essential in teeth development, blood coagulation, and intracellular cement substance integrity [37]. The mineral constitution of OMB is summarized in Figure 3. The calcium concentration of OMB was found to be 412 mg/100 g. One of the key trace elements required is iron, which is helpful in the synthesis of hemoglobin, central nervous system functioning, and oxidation of carbohydrates, fats, and proteins [38,39], which is further important in preventing diabetes. The low iron content in the body leads to infection in the gastrointestinal pathway, epistaxis (nose bleeding), and myocardial infection [40]. Figure 3 presents the iron content of OMB, which was found to be 47 mg/100 g. Zinc is another required trace element having important roles in many cellular processes such as normal body growth, development of the brain, behavioral responses, formation of bone, and smooth wound healing [41]. Protein and carbohydrate metabolism requires zinc. The hepatic stellate cells (HSCs) of the liver are the storage sites of vitamin A, and zinc is involved in its mobility to other body parts from the liver. Zinc metalloenzymes are involved in DNA and RNA biosynthesis [42]. The deficiency of zinc is commonly seen in people suffering from Crohn’s disease, hypothyroidism, and gum disease (Periodontitis). People with zinc inadequacy are vulnerable to viral infections and diabetes mellitus. Zinc plays a favorable role in treating viral infections such as AIDS, prostate gland enlargement, rheumatoid arthritis, laceration, acne, eczema, and stress [43]. Figure 3 presents the zinc content of OMB, which was found to be 16.31 mg/100 g. Other minerals detected in okra mucilage in significant amount were phosphorus (60 mg/100 g), potassium (418 mg/100 g), and sodium (9 mg/100 g). A high potassium level in the body causes an increased utilization of iron in the body. Potassium is also beneficial to patients administered with diuretics for hypertension control and those having excessive potassium excretion via body fluid [44]. 

### 2.4. Antidiabetic Activity

The use of plant-based approaches in the existing modern medications system for the treatment of chronic disease such as diabetes is gaining recognition. Majorly, two of the α-amylase and α-glucosidase enzymes are considered to be responsible for diabetic conditions: α-amylase begins the process of carbohydrate digestion by hydrolysis of 1, 4-glycosidic linkages of polysaccharides (starch, glycogen) to disaccharides, and α-glucosidase catalyzes the disaccharides to monosaccharides, which leads to postprandial hyperglycemia. Hence, inhibitors of α-amylase and α-glucosidase are useful for the control of high glucose level, as they delay carbohydrate digestion, which consequently reduces the postprandial plasma glucose level [16]. 

**Figure 4:**
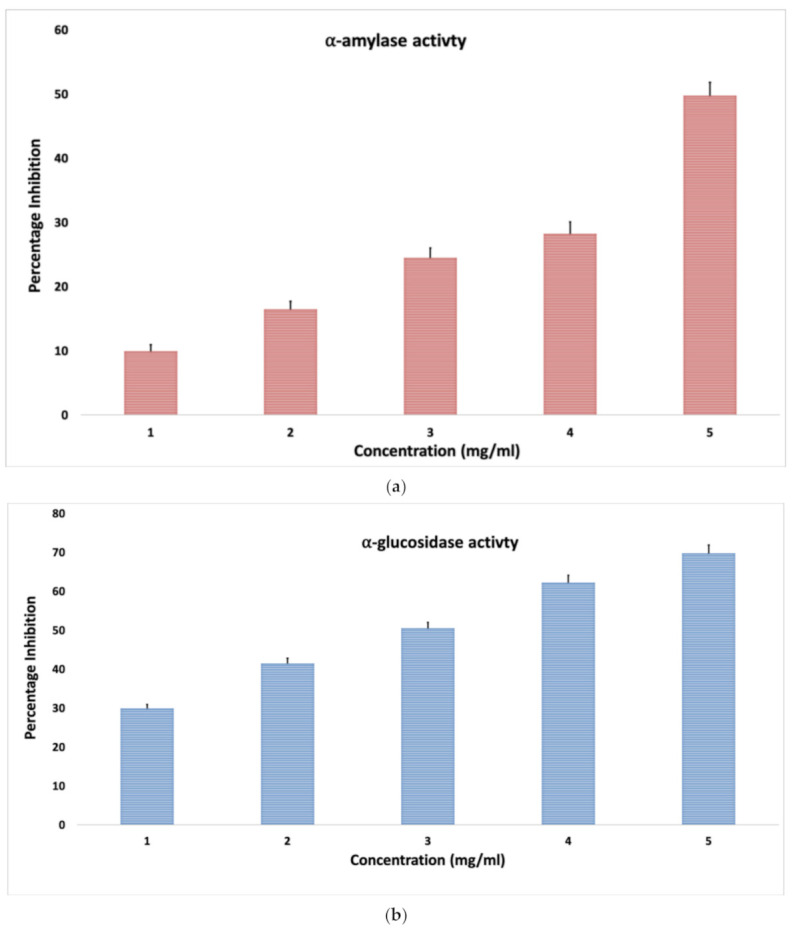
Screening of (**a**) α-amylase inhibitory assay, (**b**) α-glucosidase inhibitory assay of okra mucilage biopolymer at various concentrations. All experiments were performed in triplicate, and data are expressed as mean ±standard deviation.

To study the antidiabetic activity of OMB, α-amylase enzyme assay (Figure 4a) and α-glucosidase inhibitory assay (Figure 4b) were performed. The α-amylase inhibition enzyme assay of okra mucilage revealed that the new developed okra biopolymer had significant anti-diabetic property with the increase in the concentration of biopolymer, and its inhibitory activity was highest. On the other hand, α-glucosidase inhibitory assay showed that OMB had concentration-dependent inhibitory effects. The mucilage biopolymer at different concentration of 1, 2, 3, 4, and 5 mg/mL showed 9.9, 16.5, 24.5, 28.2, and 49.8 α-amylase inhibitory activity as well as 30, 41.5, 50.5, 62.2, and 69.7 α-glucosidase inhibitory activity, respectively. Figure 4a,b showed that the higher the concentration, the higher the inhibition of α-amylase and α-glucosidase inhibition. This could be due to high concentrations representing more solutes in the form of secondary metabolites from okra polymer, which had the ability to inhibit the action of both antidiabetic enzymes.

Earlier, the antidiabetic activity of okra has been reported by Ahmad et al. (2016), and they found that aqueous extract of okra at different increasing concentrations (50, 100, 150, 200, and 250 µg/mL) has reportedly increased the inhibition for both percentage of α-amylase and α-glucosidase enzyme. This reported result was consistent with our results [45,46]. In both the assays, the mucilage polymer showed significant results, indicating that OMB has potential, and it needs to be further explored for toxicity studies and clinical trials, and by virtue of that, it could become an important anti-diabetic agent.

## 3. Materials and Methods

### 3.1. Materials

Mature okra pods were collected during the month of September 2020 from the local markets of Hail city, Saudi Arabia. Samples collected from the local market were carefully assorted, cleaned, washed from the earthy or waste material, and further dried under the shade for 1 day. The kits used for α-amylase enzyme, α-glucosidase inhibitory assays, and ethanol was bought from Sigma-Aldrich^®^ (St. Louis, MO, USA). No additional purification was done in these kits before using them for the experiments. All other reagents used to carry out the experiments in this study were of analytical grade.

### 3.2. Extraction of Okra Mucilage

The okra pods were sliced, deseeded, and then water-soaked at ambient temperature. Liquid fraction or filtrate was separated from the solid content after 12 h using a muslin cloth. To the filtrate, triple the volume of ethanol was added. The liquid was stirred slowly by hand until the mucilage was fully precipitated. The mucilage was left to dry in an oven for about 12 h at 30 °C. Later, the dried mucilage was ground into powdered form evenly using a grinder and then passed through sieves. The finely pulverized polymer was kept in polyethylene pouch bags in the dark until further analyses [47].

### 3.3. Characterization of Okra Mucilage Biopolymer 

The FT-IR experiments were carried out using the Thermo Scientific^®^ Nicolet TM 6700 FT-IR spectrophotometer. The attenuated total reflection was performed to obtain the spectra. In this technique, Zn-Se crystals were used to press the samples, and 4000–650 cm^−1^ was the range of collection. An average of 16 scans having a resolution of 4 cm^−1^ was considered.

### 3.4. Quantitative Yield Determination

The percentage yield was calculated by the previously reported method of Jouki et al. (2014) [48,49]. The weight of okra pods without seeds in grams (m_s_) was used to calculate the yield. After the extraction, the weight of mucilage polymer was converted into milligrams (m). The percentage yield was calculated using Equation (1) as mentioned below.

(1) $$\mathrm{Yield}=\frac{m}{m_{s}}$$

### 3.5. Swelling Index

The swelling index (SI) was determined according to the method reported by Cotrim et al. (2016) with certain modifications. In a graduated cylinder of 10 mL, 0.1 g of okra mucilage biopolymer was transferred. After measuring the initial dried sample volume, ionized water was added, and a final volume of 10 mL was made. The mucilage polymer swelled up to form a viscous gel. After 24 h, the volume of swelled up mucilage was measured. The swelling index was calculated using Equation (2) mentioned below [49].

(2) $$\mathrm{SI}=\frac{V_{f}}{V_{b}}$$

where V_f_ = final volume 24 h later, and V_b_ = dry mucilage initial volume.

### 3.6. Solubility of Okra Mucilage Biopolymer

Dry okra mucilage biopolymer (0.1 g) was taken in a graduated cylinder of 10 mL to study its solubility in water. Deionized water was added to make the volume up to 10 mL, and the sample solutions were left for 6 h. Furthermore, the dispersed samples were subjected to magnetic stirring at 60 °C for 1 h. Later on, the sample solution was centrifuged at 4000 rpm for 30 min. After the centrifugation process, the insoluble matrix was separated and dried at 105 °C until it achieved constant weight. The solubility percentage of the sample was obtained as per Equation (3), as mentioned below [31]

(3) $$S=\frac{w_{1}-w_{2}}{w_{1}}\times 100$$

where S = percentage solubility, w_1_ = initial mass of dry mucilage, and w_2_ = mass of insoluble dry matter obtained after centrifugation.

### 3.7. Determination of Mineral Content

The mineral analysis of OMB was carried out according to the procedure mentioned in the AOAC (2016). Additionally, sample preparation was made by taking 1 g of the pre-dried sample in crucible and burned over a hot plate followed by ashing inside the muffle furnace at 550 °C. Once the sample cools down, it was mixed 20% supra pure nitric acid (50 mL) in an Erlenmeyer flask and heated at 70–85 °C for 6 h. Meanwhile, during the digestion of the sample, supra pure nitric acid was added intermittently to maintain the volume. Furthermore, the sample was filtered cool and the volume was made up to 100 mL in a volumetric flask using deionized water. Afterwards, the prepared samples were placed for analysis of selected elements [50]. The standard flame emission photometer (PerkinElmer, Waltham, MA, USA) was used to determine the concentrations of sodium (Na) and potassium (K) present in the okra mucilage. Phosphorus (P) content was quantified by the vanadomolybdate method AOAC (2016). Atomic absorption spectrophotometer (PerkinElmer, Waltham, MA, USA) was used to measure calcium (Ca), iron (Fe), and zinc (Zn) concentrations [51].

### 3.8. Antidiabetic Activity

To investigate the in vitro antidiabetic activity, α-amylase inhibitory assay and α-glucosidase inhibitory assay were performed.

#### 3.8.1. α-Amylase Inhibitory Assay

α-Amylase inhibitory activity of OMB was carried out as per the method presented by Oliviya et al. (2018) with slight modification. Non-identical aliquots of mucilage biopolymer (1, 2, 3, 4, and 5 mg/mL), were taken separately. Subsequently, 250 µL of 0.02 N sodium phosphate buffer containing α-amylase solution (0.5 mg/mL) was added to it. Starch solution (1%, 250 µL) prepared in 0.02 M sodium phosphate was added to the solution. The incubation of 10 min at 25 °C was provided to this mixture. With the addition of 500 µL of dinitrosalicylic acid, the reaction was terminated. Then, it was kept for 5 min in a boiling water bath. Then, the solution was cooled down, and absorbance (Abs) was recorded at 540 nm [52]. Negative control was prepared using distilled water, and the percentage inhibition was calculated by using following formula [53].

$$\%\;\mathrm{Inhibition}=\frac{\mathrm{Abs}.{\left(540\right)}_{\mathrm{control}}-\mathrm{Abs}.{\left(540\right)}_{\mathrm{sample}}}{\mathrm{Abs}.{\left(540\right)}_{\mathrm{control}}}\times 100$$

#### 3.8.2. α-Glucosidase Inhibitory Assay 

α-Glucosidase inhibitory activity of okra mucilage biopolymer was carried out as per the method presented by Oliviya et al. (2018) with slight modification. Pre-incubation of 100 µL of glucosidase with 50 µL at different concentration (1, 2, 3, 4, 5 mg/mL) of mucilage biopolymer extract were taken separately. To start the reaction, 50 µL of 3.0 mM P-nitrophenyl-α-D-glucopyranoside was added in 20 mM phosphate buffer. The solution was incubated for 20 min at a temperature of 37 °C. At this stage, the reaction was terminated by adding 0.1 M of Na_2_CO_3_. The absorbance was measured at 405 nm [52]. Negative control was prepared using distilled water, and the percentage inhibition was calculated by using the following formula [53].

$$\%\;\mathrm{Inhibition}=\frac{\mathrm{Abs}.{\left(405\right)}_{\mathrm{control}}-\mathrm{Abs}.{\left(405\right)}_{\mathrm{sample}}}{\mathrm{Abs}.{\left(405\right)}_{\mathrm{control}}}\times 100$$

### 3.9. Statistical Analysis 

The data of all the experiments were expressed as mean ±standard deviation of triplicate measurements. All the analysis were carried out using IBM SPSS software version 23.0 (IBM Corp. Armonk, NY, USA). 

## 4. Conclusions

Okra, an easily available nutritive vegetable crop, has been found very interesting in terms of mineral content. Mucilage present in okra has been found to be a rich source of polysaccharides. Our study showed that the newly developed biopolymer had key physicochemical characteristics. Key parameters such as swelling index, solubility, as well as mineral analysis indicated that the newly developed biopolymer could become a potential source for food and pharmaceutical industries as packaging materials (edible polymer), emulsion stabilizers, binding agent, water retention agent, thickeners, etc. The high swelling index of biopolymer at different pH could open the door of possibilities for the development of edible polymer as well as for the development of nutraceuticals by incorporating bioactive components. In addition, FT-IR analysis suggested that it contains polysaccharides majorly composed of galactose, rhamnose, and galacturonic acid. Furthermore, mucilage biopolymer was evaluated for in vitro antidiabetic activity, and both α-amylase and α-glucosidase inhibitory assay showed positive response against the okra mucilage biopolymer, indicating its possible application for the advancement of anti-diabetic agent. Therefore, based upon our results, we can conclude that the newly developed okra mucilage biopolymer is enriched with nutritional content as well as it could become an important natural source for various food, nutraceutical as well as pharmaceutical applications. 

## Data Availability

All data generated or analyzed during this study are included in this article.

## Sample Availability

Samples of the compounds are not available from the authors.
